# Transcriptome Profile in Hippocampus During Acute Inflammatory Response to Surgery: Toward Early Stage of PND

**DOI:** 10.3389/fimmu.2019.00149

**Published:** 2019-02-05

**Authors:** Xuwu Xiang, Yang Yu, Xiaodong Tang, Manli Chen, Yueying Zheng, Shengmei Zhu

**Affiliations:** Department of Anesthesiology, The First Affiliated Hospital, College of Medicine, Zhejiang University, Hangzhou, China

**Keywords:** cognitive dysfunction, surgery, hippocampus, neuroinflammation, RNA-Seq

## Abstract

Perioperative neurocognitive disorders (PND) are common complications observed in surgical patients, but there are no effective treatments and the detailed mechanisms remain largely unknown. In this study, transcriptome analysis was performed to investigate the hippocampal changes after surgery and underlying molecular mechanisms of PND. Tibial fracture surgery was performed in 3–4 months old C57BL/6J mice to mimic human orthopedic surgery. We demonstrated that memory consolidation of the hippocampal-dependent trace-fear conditioning task was significantly impaired. By using ELISA, a significant elevated IL-6 was observed both in circulating system and central nervous system and peaked at 6 h post-surgery, but transiently returned to baseline thereafter. Hippocampus were collected at 6 h post-surgery then processed for RNA-Seq. A total of 268 genes were screened differentially expressed between the Surgery and Control group, including 170 up-regulated genes and 98 down-regulated genes. By functional enrichment analysis of differently expressed genes, several KEGG pathways involved in inflammatory mediator regulation of TRP channels, neuroactive ligand-receptor interaction and cholinergic synapse were overrepresented. Quantitative real-time PCR confirmed 15 dysregulated genes of interest. These results provide a comprehensive insight into global gene expression changes during the acute presence of hippocampal inflammation and a better understanding on early stage of PND.

## Introduction

Cognitive impairments are frequent complications among surgical patients. These impairments are now termed as Perioperative neurocognitive disorders (PND), including acute postoperative delirium and long-lasting postoperative cognitive dysfunction (1). PND are becoming increasing concerns as they are associated with various adverse outcomes, including prolonged hospitalization, increased complications and mortality, and decreased quality of life (2, 3).

Surgery trauma itself provokes release of damage-associated molecular patterns (DAMPs), leading to innate immune activation and increasing proinflammatory cytokines, both in peripheral and central. Appearance of inflammatory cytokines in cerebral spinal fluid (CSF) has been repeatedly observed in surgical patients (4–8). Additionally, in a remarkable clinical study, a profound immune activity change in the human brain after surgery was found by positron emission tomography (PET) and associated with their performance on mental test (9). Growing preclinical data from rodent models suggest neuroinflammation, which featured as proinflammatory cytokines accumulation and glial activation in hippocampus, plays a key role in the pathogenesis and progression of PND. Increased proinflammatory cytokines serve as immune-to-brain signaling which are capable of disrupting synaptic plasticity (10), a neurochemical foundation of learning and memory (11). Preserving cognitive function can be achieved by attenuating the inflammatory signaling pathways (12, 13). Accumulating evidences suggest that interleukin-6 (IL-6), one of the main elevated proinflammatory cytokines in PND, plays a critical role in cognitive decline after surgery (14–16).

Hippocampus is widely recognized as playing important roles in learning and memory which is also vulnerable to neuroinflammation after surgery. Several studies have reported deficits in trace-fear conditioning (TFC) test after structure lesions that input into hippocampus or blocking receptor functions in hippocampus by pharmacological agents (17–20), indicating that it is hippocampal-dependent. Moreover, TFC test correlates well with performance in a Morris water maze (21), a well-validated assay of learning and memory often used in clinical models of cognitive dysfunction. Therefore, TFC test assessed in rodents within days following surgery has been widely used in PND studies (14, 22).

Most previous studies focused on the long-term effects of PND, little is known about early events of PND. In fact, administration of LPS causes synaptic transmission change as early as 2 h, which is mediated by rapid response of pericytes (23). Gene expression profiling in brain tissue during acute stage could provide a more comprehensive insight into the pathophysiology of PND.

In this study, we determined perioperative dynamic levels of IL-6 and applied high-throughput RNA-Seq technology to identify dysregulated genes during the acute presence of neuroinflammation. We also employed functional gene set enrichment to identify classes of differentially regulated genes which may associate with PND. Finally, we used quantitative real-time PCR (qRT-PCR) to confirm expression of 15 dysregulated genes of interest. These findings may further our understanding of biological mechanisms of PND, especially during the early stage of the disease.

## Materials and Methods

### Animals

Three to four months old male C57BL/6J mice were purchased from Shanghai SLAC laboratory animal Co., Ltd. (Shanghai, China). All animal experiments were carried out with the approval of the Animal Care Committee at Zhejiang University. Mice were housed in polypropylene cages and maintained at 25°C under reverse phase 12 h light-dark cycle with *ad libitum* access to water and rodent chow. Animals were tagged and randomly allocated to each group before any procedure (For RNA-seq and qRT-PCR, 4 mice were used in control and surgery groups; for ELISA, 5–8 mice were used in control and surgery groups).

### Experiment Paradigm

**Figure 1:**
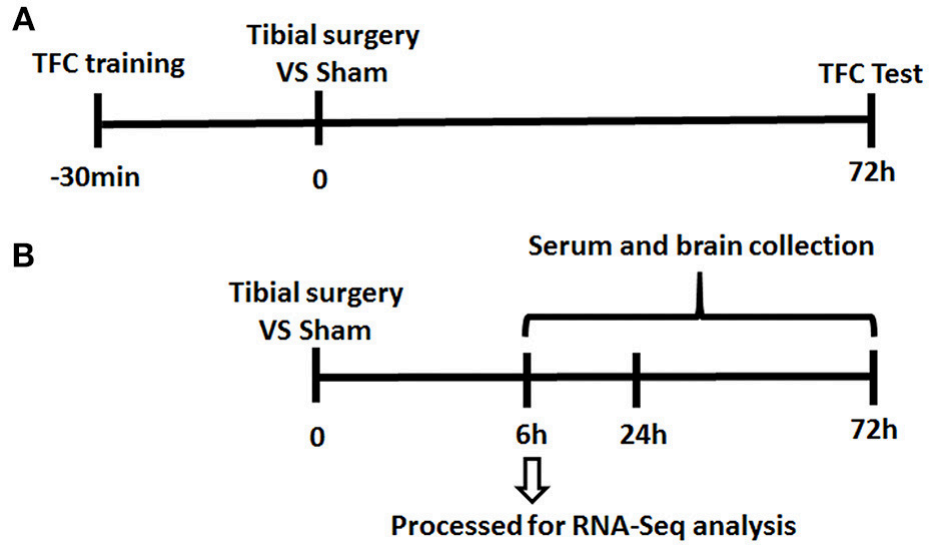
Experiment Paradigm. Mice were divided into 2 cohorts. **(A)** One cohort for behavior experiment includes the training session of the memory test performed 30 min before tibial fracture and TFC test session; **(B)** the other cohort for neuroinflammation assessment complete tibia surgery without TFC training and test session and collect serum/ tissue at time point of 6/24/72 h.

Since almost undetectable IL-6 changes were observed by ELISA at 3 days post-surgery, at the time when TFC test was performed, mice were divided into 2 separate cohorts (presented in Figure 1).

### Tibial Fracture Surgery

Surgery was consisted of an aseptic open tibial fracture with intramedullary fixation performed under general anesthesia, including 5% sevoflurane (Sevorane™, Abbott, Switzerland) in 500 ml/min O_2_ using a rodent inhalation anesthesia apparatus (MIDTRX VIP2000, Midmark, Dayton, OH, USA) and Tramadol (Merck & Co., Inc., Kenilworth, NJ, USA) as previously described with minor modifications (22, 24). Briefly, the left hind paw was disinfected. A median paw incision was then performed, followed by the insertion of a 28 G needle (~0.38 mm) in the tibial intramedullary canal. Then osteotomy was performed at the junction of the middle and distal thirds of the tibia. The fixation needle remained *in situ* and be cut flush with the tibial cortex. After producing the fracture, skin was closed with 5–0 Vicryl sutures (Monocryl; Ethicon Inc, Somerville, NJ); thereafter, animals were allowed to recover spontaneously from the anesthetic. Analgesia (s.c., Tramadol, 30 mg/kg) was administered after anesthetic induction and before skin incision. Control (sham-treated) mice received anesthesia and analgesia.

### Trace-Fear Conditioning Test

TFC test was used to assess learning and memory as previously published (22). Freezing behavior is an indicator of aversive memory which is evoked by contextual cues related to a fear-inducing stimulus-response pairing learned previously. The extent of this learning (contextual testing) was recorded in a TFC chamber (Shanghai XinRuan Information Technology Co., Ltd, Shanghai, China). Memory impairment is indicated by a decrease in freezing time.

#### Training

On the training day, mice were placed in the TFC chamber and allowed to explore it for 100 s. Mice were then exposed to an auditory cue (75–80 Db, 5 kHz, conditional stimulus) for 20 s, followed 20 s later by a 2-s foot shock (0.8 mAmp; unconditional stimulus). The tone and foot-shock pairing was repeated with an inter-trial interval of 100 s. After training is completed, allow the mice to remain in the testing chamber for 1 min before removing.

#### Contextual Testing

Three days after the training session, the mice were placed back into the same TFC chamber but without any tone or shock for 5 min. Video tracking system recorded the time spent freezing as a fraction of total time in the chamber.

### Cytokine Measurement

Blood was transcardially collected, then perfused with saline and hippocampus were then rapidly extracted, stored at −80°C until assayed. Blood samples were centrifuged at 3,000 rcf per minute for 15 min and serum were collected and stored at −20°C until assayed. ELISA assays (MultiSciences, Hangzhou, China) were performed to measure IL-6 levels in the serum and hippocampus, following manufacturer's instructions.

### RNA-Seq Analysis

Total RNA was extracted from hippocampus of mice in the Control and Surgery groups (four mice in each group) using TRIzol Reagent (Invitrogen) and treated with DNase I at 37°C for 20 min to digest DNA. Then, the mRNA was purified using poly-T oligo-attached magnetic beads and followed by fragmented into small pieces using divalent cations under elevated temperature. The cleaved RNA fragments were copied into first strand cDNA using reverse transcriptase and random primers. Then second strand cDNA synthesis was synthesized by using DNA polymerase I and retained mRNAs were removed by RNase H. These cDNA fragments then have the addition of a single “A” base and subsequent ligation of the adapter. Sequencing was performed on the BGISEQ-500RS developed by BGI Co., Ltd. (China). The raw RNA-Seq data were filtered into clean reads, followed by mapping to the mouse reference genome (mm10) using HISAT. The gene quantification was analyzed using RSEM quantification tool, and the expression level of each gene was calculated in fragments per kilobase of exon per million fragments mapped. The NOISeq method was used to screen the differentially expressed genes (DEGs) between the control and surgery groups according to the criteria of fold change ≥ 2 and diverge probability ≥ 0.8. The gene ontology (GO) annotation mapped all DEGs to GO terms in the database (http://www.geneontology.org/). The public KEGG database was used to perform the pathway enrichment analysis (25).

### qRT-PCR

qRT-PCR was performed on a CFX 96 real-time PCR thermocycler (Bio-Rad Laboratories, Hercules, CA, USA) using same RNA samples used for RNA-Seq. Primer sequences were listed in supplemented file Table S1. For amplification, SYBR Premix Ex Taq II (Takara Bio Inc., Otsu, Japan) was used as instructed in the manufacturer's manual. Relative changes of mRNA expression were analyzed with the 2^−ΔΔ*Ct*^ method and GAPDH was serving as an internal reference. These standardized data were used to calculate fold changes in gene expression. All real-time PCR amplifications were performed in triplicate.

### Statistics

qRT-PCR and ELISA data were expressed as means ± standard error of the mean (SEM). Graphical and statistical analyses were performed using the Prism 6.0 (GraphPad Software Inc., CA, USA). Comparisons between two groups were performed using two-tailed Student's *t*-test, assuming equal variance. *P* < 0.05 was considered significant.

## Results

### Transient Period of IL-6 Elevation After Surgery

To investigate the mechanisms of PND, an aseptic tibial fracture with internal fixation surgery model was established and memory of association learned preoperatively was assessed in a trace-fear conditioning (TFC) paradigm (Figure 1).

**Figure 2:**
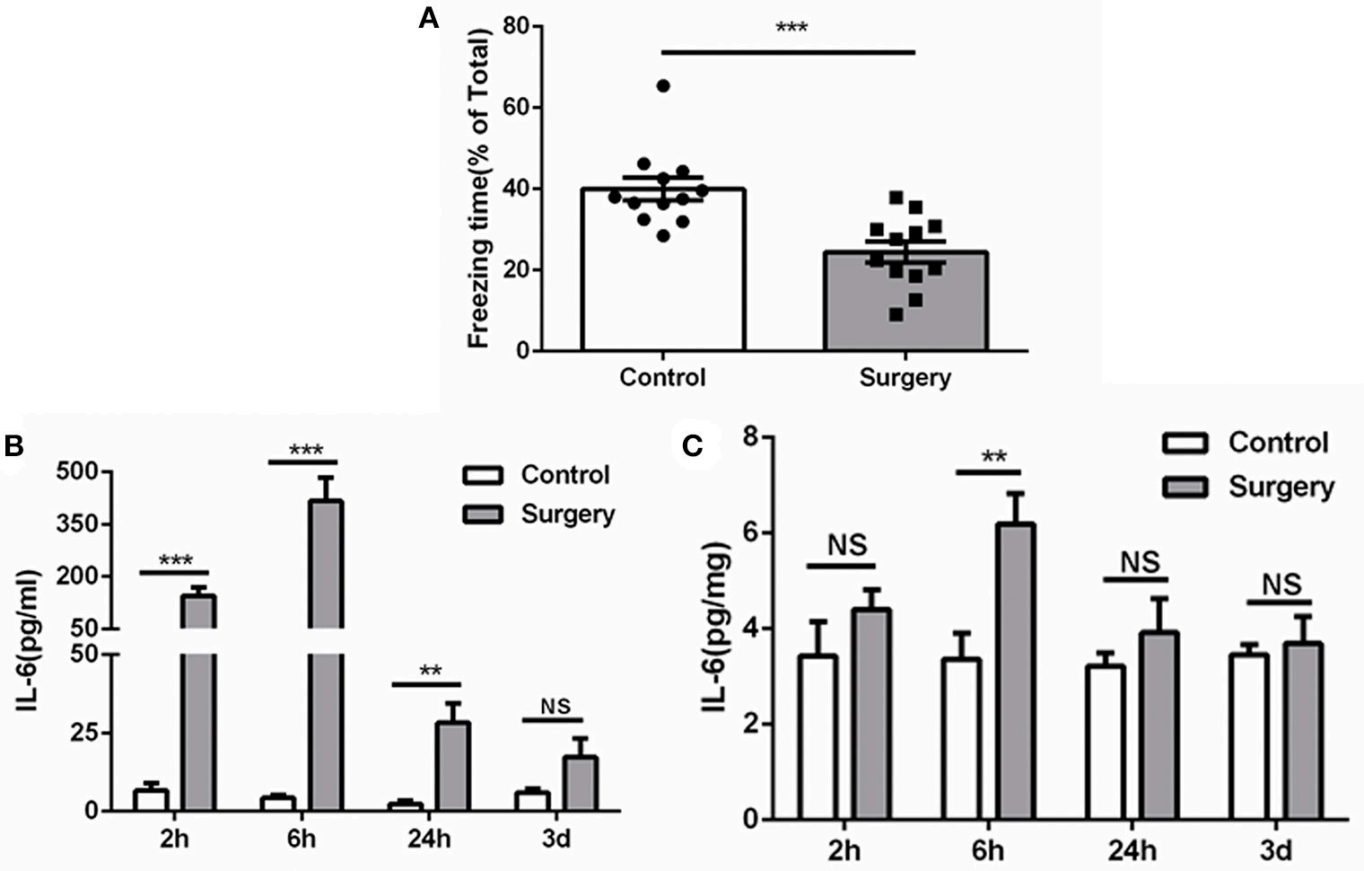
Surgery induces cognitive decline and inflammation. **(A)** Freezing in response to contextual cues, showed as a percentage of total time in the testing environment prior to surgery. **(B)** Serum ELISA, showing the significant elevated IL-6 level after surgery in serum. **(C)** Tissue ELISA, showing the postoperative elevated IL-6 level in hippocampus (*P* < 0.01 at 6 h). Data represent mean ± SEM; *n* = 5–8/group. NS, no significant; ^***^P < 0.001; ^**^P < 0.01 for Surgery vs. Control.

Compared with anesthesia-matched sham procedure, Surgery group showed a significant reduction of freezing time at 72 h (Figure 2A), indicating cognitive decline was induced by surgery. Meanwhile, IL-6 was increased promptly post-surgery both in serum and hippocampus and reached a plateau at 6 h, then falls thereafter (Figure 2B). These findings consistent with previous studies (22, 26) and demonstrate a mild and transient period of hippocampal inflammation after surgery.

### Transcriptome Profile in Hippocampus

To gain a more comprehensive insight into the gene expression changes of hippocampus during the early presence of neuroinflammation, RNA-Seq and DEGs screening analyses were performed on 8 samples (four mice in Control and Surgery group, respectively).

**Figure 3:**
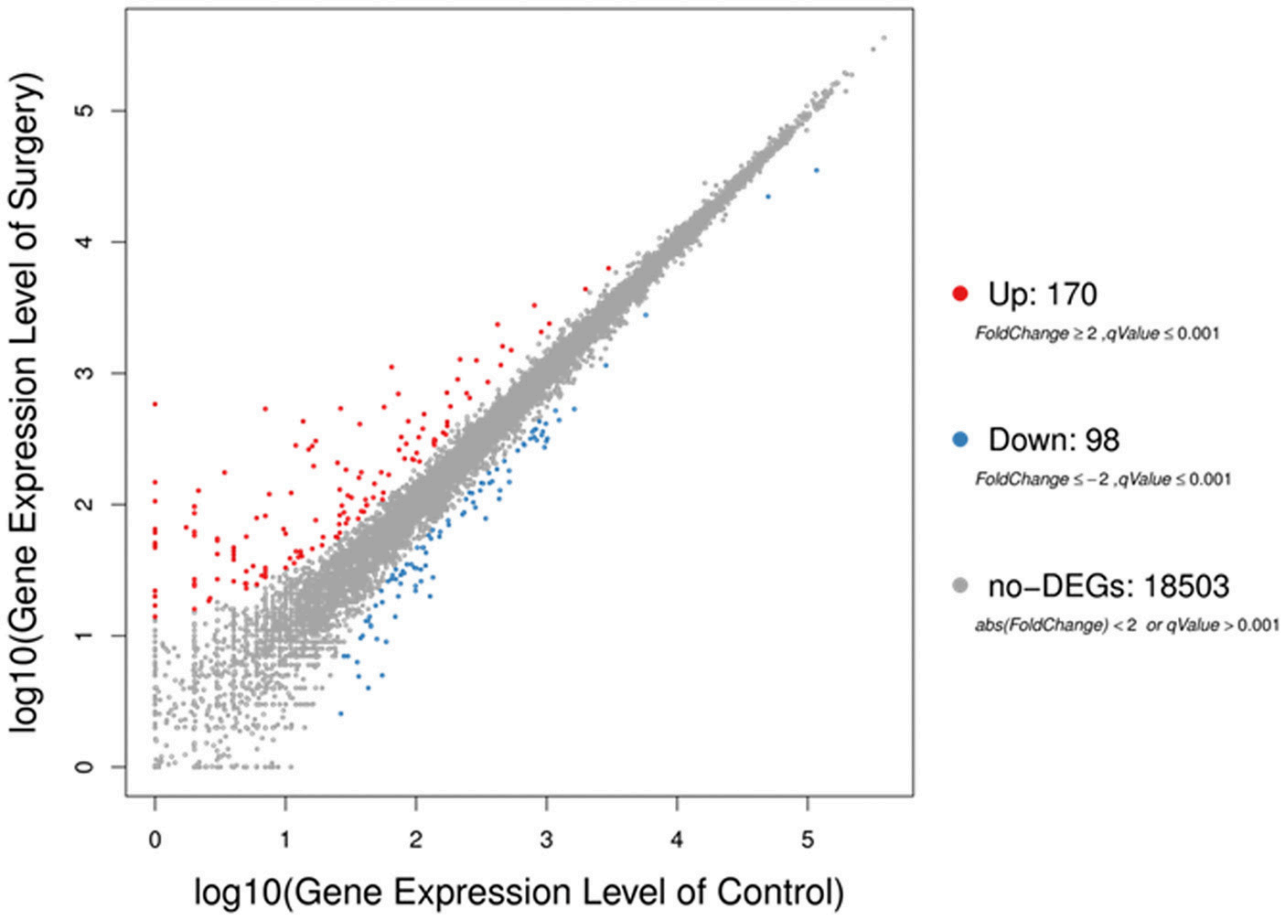
Scatter plots of all expressed genes in each pairwise. Blue represents downregulated genes, orange represents upregulated genes and brown represents non-regulated gene. Fold change ≥2, *q*-value ≤ 0.001 used as screening threshold.

The sequencing generated, on average 21.69 M clean reads and mean clean reads ratio of 98.87%. Of 2,05,782 genes in the mice reference genome, 18,771 mRNAs were identified; of those, 268 genes were differentially expressed following surgery (Table S2). The majority (170) were increased (Fold Change ≥ 2, *q*-Value ≤ 0.001), while 98 were decreased (Fold Change ≤ 2, *q*-Value ≤ 0.001) (Figure 3).

### Gene Ontology (GO) and Enriched KEGG Pathway Analysis

**Figure 4:**
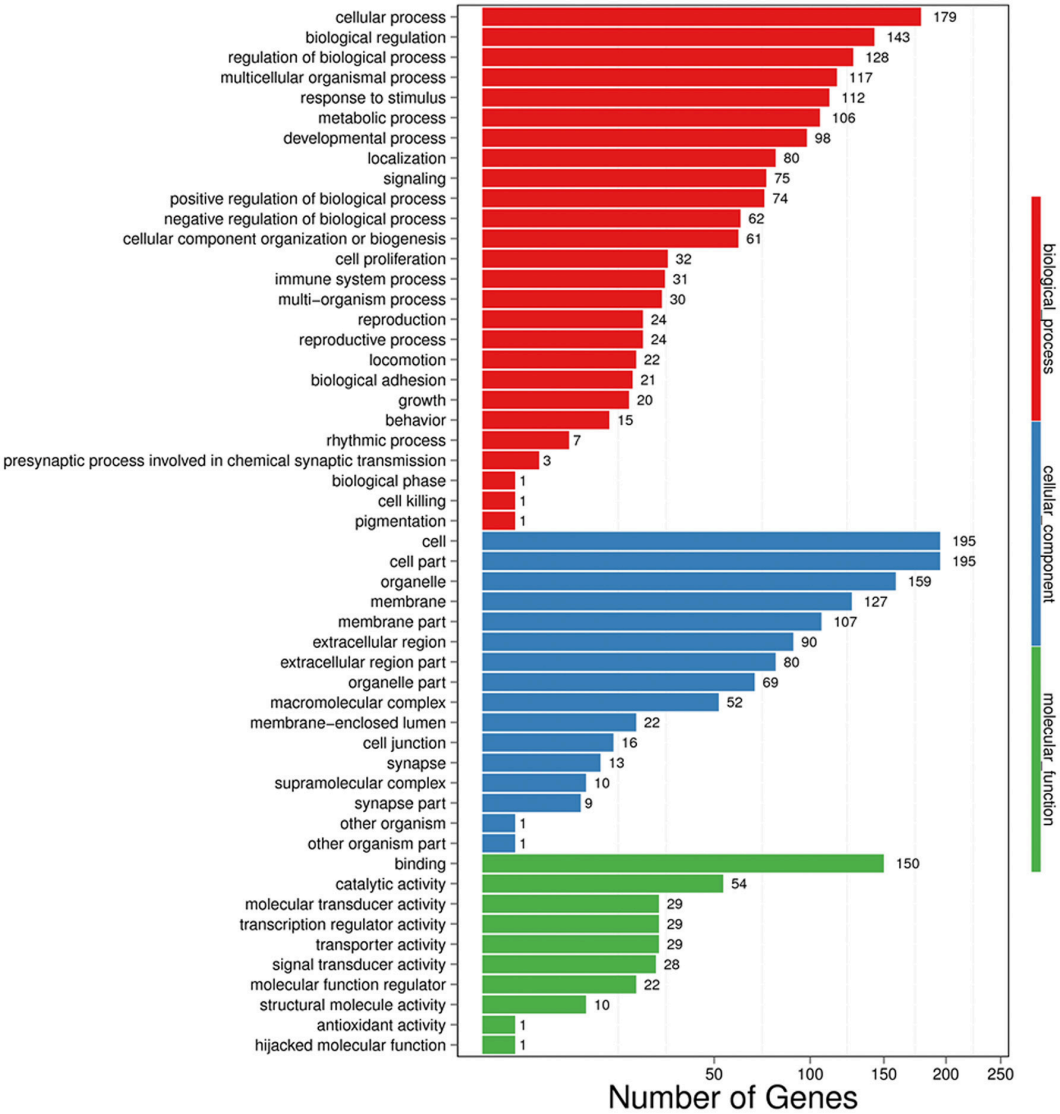
GO functional classification on DEGs for each pairwise. X-axis indicates the number of DEGs (the number is presented by its square root value). Y-axis represents GO terms. All GO terms are grouped into three ontologies: blue is for biological process, green is for cellular component and red is for molecular function.

In GO analysis, DEGs were classified into biological process (BP), cellular component (CC), and molecular function (MF). Among BP classes, cellular process (179 genes) contained the largest group of genes, and genes involved in biological regulation constituted the second largest group with 143 genes. The third largest group included 128 genes involved with regulation of biological process. Additionally, 117 genes were identified that were involved in multicellular organismal process, 112 genes were also identified involved in response to stimulus (Figure 4).

In the CC category, the genes expressed were mostly enriched for cell (195 genes), cell part (195 genes), and organelle (159 genes). Additional DEGs were related to membrane (127 genes), membrane part (107 genes), extracellular region (90 genes), extracellular region part (80 genes), organelle part (69 genes), and macromolecular complex (52 genes) (Figure 4).

Finally, in MF classes, binding (150 genes) and catalytic activity (54 genes) were top two enriched terms. Additional DEGs were involved in molecular transducer activity (29 genes), transcription regulator activity (29 genes), transporter activity (29 genes), and signal transducer activity (28 genes), suggesting an active and vigorous response provoked in hippocampus (Figure 4).

**Figure 5:**
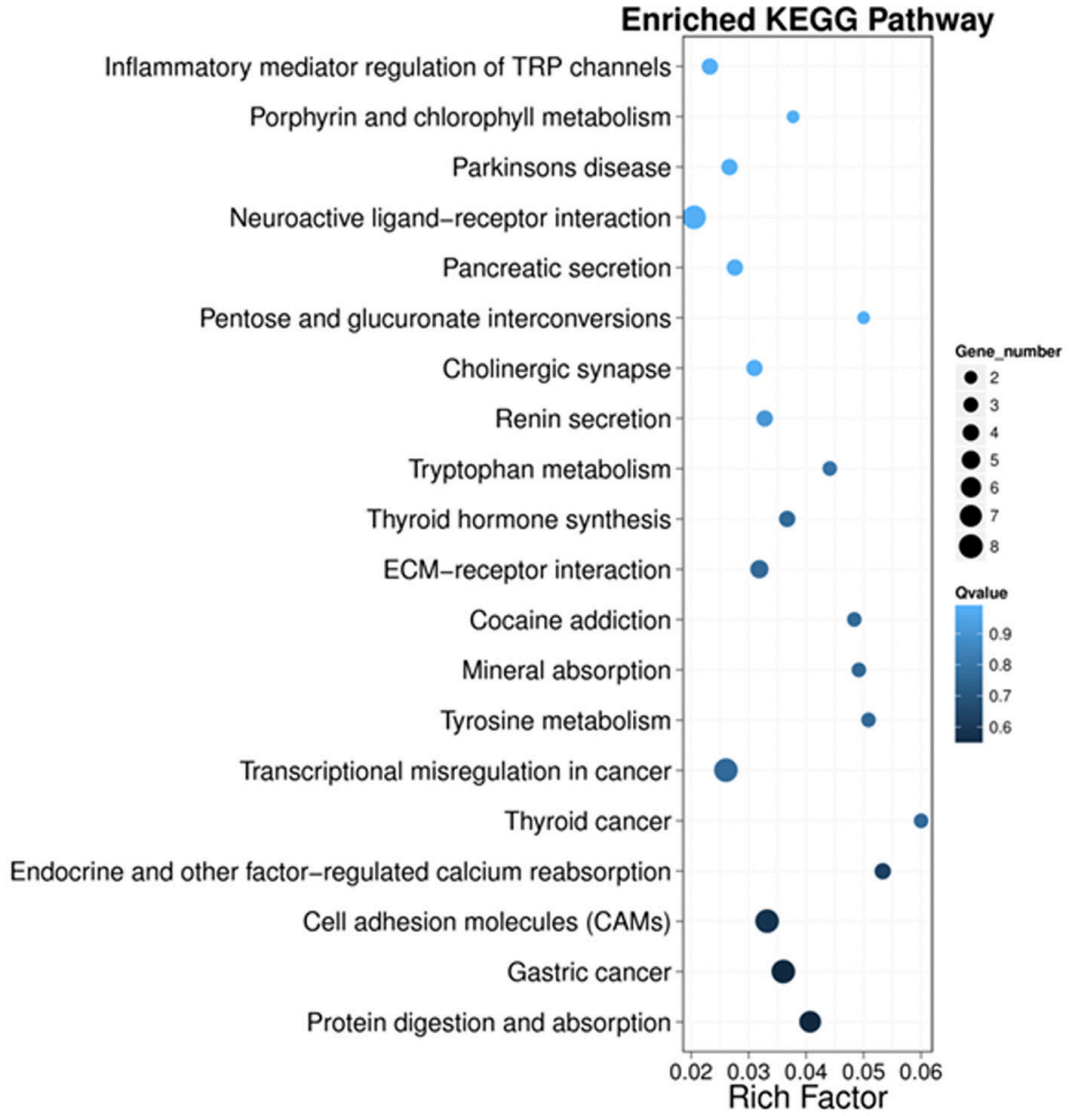
Scatter plot of the top 20 KEGG enrichment results of DEGs in each pairwise comparison. Rich Factor is the ratio of DEG numbers annotated in this pathway term to all gene numbers annotated in this pathway term. A large Rich Factor represents greater intensiveness. The *Q*-value ranges from 0 to 1, and a lower *Q*-value represents greater intensity.

Next, we performed KEGG pathway enrichment analysis to further understand gene biological functions of DEGs. Surprisingly, several neurogenic disease-associated pathways were identified to be in the top 20 enriched pathway terms, including neuroactive ligand-receptor interaction, cholinergic synapse, Parkinson's disease, inflammatory mediator regulation of TRP channels and cocaine addiction (Figure 5). Moreover, some other pathways involved in cell communication or metabolism were also displayed (Figure 5), such as cell adhesion molecules (CAMs), tyrosine metabolism, ECM-receptor, tryptophan metabolism, pentose and glucuronate interconversions, and porphyrin and chlorophyll metabolism.

### Validation of RNA-Seq Results by qRT-PCR

**Figure 6:**
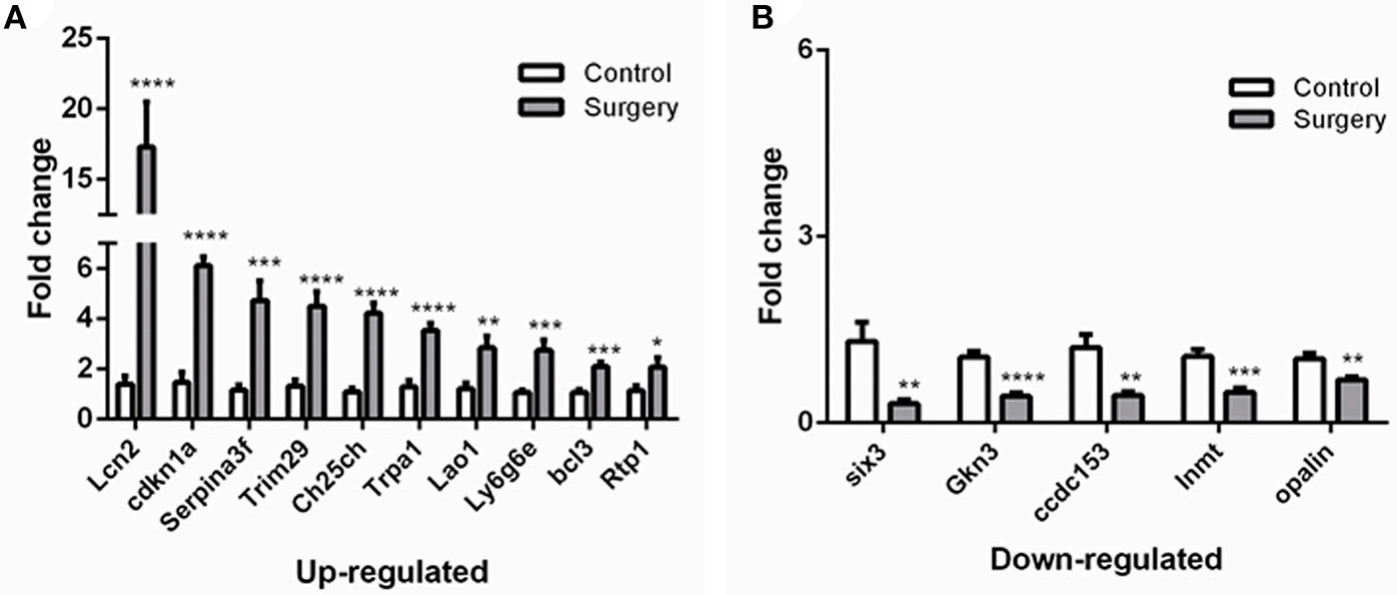
qRT-PCR validation of selected DEGs identified by RNA-seq analyses.**(A)** Relative expression of up-regulated genes. **(B)** Relative expression of down-regulated genes. After normalized to GAPDH, fold changes were calculated by comparing the normalized copy number in each sample to the mean of Control group. Data represent mean ± SEM; *n* = 4/group. ^*^P < 0.05; ^**^P < 0.01; ^***^p < 0.001; ^****^p < 0.0001.

To verify the results of RNA-Seq analyses, 15 DEGs of interest were selected for qRT-PCR (Table 1). Gene expressions measured by qRT-PCR exhibited similar changes to the gene expression analyses (Figure 6), with 10 genes (Lcn2, Cdkn1a, Serpina3f, Trim29, Ch25ch, Trpa1, Lao1, Ly6g6e, Bcl3, Rtp1) up-regulated and 5 genes (Six3, Gkn3, Ccdc153, Inmt, Opalin) down-regulated. Among these genes, Ch25h, Trpa1, Cdkn1a, Ly6g6e, Six3, and Opalin are potentially associated with neurological diseases; Lcn2, Serpina3f, Lao1, Trim29, bcl3, and Gkn3 are associated with inflammatory response. Other genes of interest for the qRT-PCR analysis included Rtp1, Ccdc153 and Inmt, which are involved in metabolism and signal transduction and showed large up- or down-regulation by both RNA-Seq and qRT-PCR.

**Table 1:** Functional properties of genes selected for qRT-PCR analysis.

**Gene ID**	**Symbol**	**log2Ratio**	**Up/Down-regulation**	**Pathway and related function**
16819	Lcn2	3.29	Up	Immune system; IL-17 signaling pathway
238393	Serpina3f	2.63	Up	Serine protease inhibitor
12642	Ch25h	1.74	Up	Lipid metabolism; risk factor of AD; contributes to cerebral Inflammation.
277328	Trpa1	1.71	UP	Inflammatory mediator regulation of TRP channels; contributes to AD developments.
100470	Lao1	1.62	Up	Amino acid metabolism.
72169	Trim29	1.40	Up	Negatively regulates innate immunity.
239766	Rtp1	1.33	Up	Receptor transporter.
12575	Cdkn1a	1.22	Up	Signaling transduction; cell growth and death.
12051	bcl3	1.17	Up	Signal transduction; TNF signaling pathway.
70274	Ly6g6e	1.05	Up	nAChR signaling.
20473	Six3	−1.94	Down	Wnt signaling; brain development.
68888	Gkn3	−1.43	Down	Immune system; NOD-like receptor signaling pathway.
270150	Ccdc153	−1.29	Down	/
21743	Inmt	−1.25	Down	Amino acid metabolism.
226115	Opalin	−1.00	Down	oligodendrocytic myelin protein; regulation of the oligodendroglial actin cytoskeleton.

## Discussion

In clinical practice, 10–26% of patients retain subtle but persistent learning and cognitive deficits, referred as PND (1, 27), resulting in higher risk for further morbidity and permanent dementia (28). In this study, we determined dynamic levels of IL-6 in serum and hippocampus, then isolated hippocampus for RNA-Seq to investigate the differentially expressed gene signature when IL-6 reached at highest level post-surgery. A total of 268 genes were screened differentially expressed, including 170 up-regulated genes and 98 down-regulated genes (Surgery vs. Control group). Gene ontology (GO) and KEGG pathway enrichment analyses were performed to explore the principal functions of dysregulated genes. It is the first report to comprehensively analyze gene expression profile during acute phase of hippocampal inflammation in PND, and may lead to further investigations on the mechanisms and resolutions of PND.

IL-6 is a late-phase proinflammatory cytokine, and its circulating level negatively correlates with cognitive performance in humans (29, 30). It consistently elevates in both the circulating and CNS following surgical trauma in rodents (14) and surgical patients (8, 31). Administering exogenous IL-6 inhibits mitogen-activated protein kinase ERK and Long-term potentiation (LTP) in a hippocampal slice (32–34), ultimately impairs cognitive performance (14). Surgery-induced decline in cognitive function could be attenuated by blocking IL-6 signals or rescued in IL-6 deficient mice (14–16). These findings demonstrated that dysregulated IL-6 levels play a key role in hippocampal inflammatory responses that negatively affect cognitive processing.

Hippocampus plays important roles in consolidation of information from short-term memory to long-term memory, and it is particularly susceptible to the effects of inflammation (35, 36). In fact, hippocampus is one of the few areas of the CNS outside the hypothalamus that expresses high levels of IL-1β, IL-6, and TNF-α receptors (37, 38). Consistent with this enhanced sensitivity, peripheral surgery has been shown to alter neuroglial metabolic coupling (39) and neurotransmission in hippocampus (26, 39).

In the present study, we employed RNA-Seq technology to analyze gene expression across the entire mice genome to find differentially expressed genes during early stage of PND. A number of differential expressed genes were identified by DEGs analysis. These genes are involved in various functions, including cellular process, biological regulation and regulation of biological process. Though lots of them contribute to neural functions in various neurodegenerative disease models, most of their dysregulations are revealed for the first time in PND. For example, Lcn2, one of the most up-regulated genes, given its features as an iron regulator and inflammatory protein in the CNS, has emerged as a new player in neurodegenerative diseases (40). Investigations into these genes' expression changes and functions would greatly improve our understanding for pathophysiology of PND.

In addition, KEGG pathway analysis of DEGs showed potential underlying mechanisms of PND, including neuroactive ligand-receptor interaction, cell adhesion molecules (CAMs), cholinergic synapse, inflammatory mediator regulation of TRP channels, and ECM-receptor interaction were severely affected. Some of them are critical for neuronal transmission, and dysfunction of these pathways may ultimately lead to disrupted neuronal plasticity and memory deficits. Specially, TRP channels can be activated by thermal and chemical stimuli and play a central role in pain sensation in peripheral nervous system (41), but it has also been proved that some of TRP channels may induce Long-term depression (LTD) in hippocampus (42), the opposite of LTP. Moreover, neuroactive ligand-receptor interaction which mediates the transduction of signals from the extracellular environment into cells may be involved in modulation of synaptic plasticity. Besides, cholinergic neurotransmission acts as a signal-to-noise ratio modulator of sensory and cognitive inputs, thus plays an important role in regulating the cognitive functions of the brain (43, 44). Dysfunction of cholinergic neurotransmission has been implicated in neurological disorders such as Alzheimer's disease, and attempts have been made to use cognitive enhancer such as acetylcholinesterase inhibitors to combat dementia (43–45). Additionally, several pathways associated with metabolism such as tyrosine metabolism and tryptophan metabolism; several other pathways involved in neurogenic diseases, such as cocaine addiction and Parkinson's disease, were also affected. These results suggest that brain functions are greatly disturbed during surgery induced hippocampal inflammation and provide a basis to understand the underlying mechanisms of PND.

Our study also has some limitations. First, in addition to IL-6, other cytokines such as TNF-α, MCP-1, and IL-1β, as well as influx of inflammatory cells into the brain parenchyma and glial activation, all contribute to cognitive deterioration after surgery, thus it would be important to fully assay all these changes. Second, we did not test permeability of blood brain barrier (BBB) at early stage of PND. BBB protects the neural microenvironment with low permeability by tight junction and low rates of transcytosis. Previous study showed increased BBB permeability and expression changes of tight junction molecules after surgery (15), yet transcellular path has not been stressed. In fact, accumulating evidences suggested impairment of BBB was a stepwise process. In stroke, rates of transcytosis were increased as early as 6 h, while profound structural defects of tight junction displayed after 2 days (46, 47). The future studies should assess the kinetics changes of BBB by using tracer molecule and TJs-labeled transgenic mice in PND. Besides, RNA-Seq technology also has some disadvantages, including an inherent lack of spatial information. Applying *in situ* hybridization (ISH) would allow us to further obtain temporal and spatial information about gene expression (48).

In conclusion, we employed RNA-Seq analyses as an unbiased approach to screen differentially expressed genes in hippocampus tissues from Surgery and Control mice 6 h post-surgery. Two hundred and sixty eight genes were screened differentially expressed, including 170 up-regulated genes and 98 down-regulated genes. Additionally, 15 dysregulated genes of interest were validated by qRT-PCR. While clear associations between these genes and cognitive behavior phenotype in PND are not yet elucidated, still, in respect of the dysregulated genes, may lead to important advances in our understanding of how early immune-related events can influence cognition and memory processes.

## Ethics Statement

This study was carried out in accordance with the National Institutes of Health guidelines and regulations. The protocol was approved by the Animal Care Committee at Zhejiang University.

## Author Contributions

XX, YY, XT, MC, YZ, and SZ conceived and designed the experiments. XX, YY, and XT performed the experiments. XX, YY, XT, MC, YZ, and SZ analyzed the data. XX and SZ wrote the paper. All the authors have read and approved of the final manuscript.

### Conflict of Interest Statement

The authors declare that the research was conducted in the absence of any commercial or financial relationships that could be construed as a potential conflict of interest.

## Supplementary Material

Table S1 Primers used in this study for qRT-PCR.

Table S2 DEGs screened between Surgery and Control groups.
