# Supplementary material for: Transcriptome Profile in Hippocampus During Acute Inflammatory Response to Surgery: Toward Early Stage of PND
Source: Front Immunol. 2019 Feb 5;10:149. doi: 10.3389/fimmu.2019.00149 (PMC6370675; doi:10.3389/fimmu.2019.00149)
Supplement: Table S1 — Primers used in this study for qRT-PCR. [file Table_1.DOCX]

**Table S1 Primers used in this study for qRT-PCR**

| **Gene ID** | **Gene symbol** | **Primer sequence（5’-3’）** |
| --- | --- | --- |
| 16819 | *Lcn2* | (F): GCAGGTGGTACGTTGTGGG  (R): CTCTTGTAGCTCATAGATGGTGC |
| 12575 | *Cdkn1a* | (F): CCTGGTGATGTCCGACCTG  (R): CCATGAGCGCATCGCAATC |
| 238393 | *Serpina3f* | (F): GGAGTATGCTTTTCCCCAAGTG  (R): GCAGACAGACCTATATTCAGGCA |
| 72169 | *Trim29* | (F): AGAATGGCACTAAAGCAGACAG  (R): AAATAGGCCACTCTTCCCCTC |
| 12642 | *Ch25ch* | (F): TGCTACAACGGTTCGGAGC  (R): AGAAGCCCACGTAAGTGATGAT |
| 277328 | *Trpa1* | (F): GTCCAGGGCGTTGTCTATCG  (R): CGTGATGCAGAGGACAGAGAT |
| 100470 | *Lao1* | (F): CTCCCCACTGTCTAAGCGTG  (R): CAAGATGGTTACCTCGTGACC |
| 70274 | *Ly6g6e* | (F): TACTGGTCACGGTCCTACTCT  (R): GGCAGCATTGCATAGGTCCT |
| 12051 | *Bcl3* | (F): CCGGAGGCCCTTTACTACCA  (R): GGAGTAGGGGTGAGTAGGCAG |
| 239766 | *Rtp1* | (F): GCTGCCCTGCCTTACACTTAC  (R): CACCTGTGGTCACACTCTTAC |
| 20473 | *Six3* | (F): TCAACAAACACGAGTCGATCC  (R): TGGTACAGGTCGCGGAAGT |
| 68888 | *Gkn3* | (F): AGACAACATGAGACGCCTTATTG  (R): CTGTCGCTAGTGTTCGTCAGC |
| 270150 | *Ccdc153* | (F): GGGAGGCTAAGACAGCGAAG  (R): GTGAGGCTTGACCACTCTCAG |
| 21743 | *Inmt* | (F): GCAGAGCAGGAAATCGTAAAGT  (R): GGGGTGTAGTCAGTGACAATGAT |
| 226115 | *Opalin* | (F): TTTACACTGCCATCGAATACGAC  (R): GCCAGTCCAATAGAGGGACCA |
| 14433 | *Gapdh* | (F): AGGTCGGTGTGAACGGATTTG  (R): TGTAGACCATGTAGTTGAGGTCA |
